# Causes of Intensive Care Unit Admissions in Children with SARS-CoV-2: A Single-Centre Observational Study

**DOI:** 10.3390/children10010075

**Published:** 2022-12-30

**Authors:** Lukáš Homola, Jozef Klučka, Dominik Fabián, Petr Štourač, Josef Šikula, Eva Vávrová, Barbora Jeřábková, Martin Sihlovec, Václav Musil, Klára Španělová, Patricia Mužlayová, Pavlína Danhofer

**Affiliations:** 1 Department of Pediatric Infectious Diseases, University Hospital Brno, Černopolní 22a, 61300 Brno, Czech Republic; 2 Faculty of Medicine, Masaryk University, Kamenice 5, 62500 Brno, Czech Republic; 3 European Reference Network on Rare Respiratory Diseases Centre, University Hospital Brno, Jihlavská 20, 62500 Brno, Czech Republic; 4 Department of Paediatric Anaesthesiology and Intensive Care Medicine, University Hospital Brno, Černopolní 9, 61300 Brno, Czech Republic; 5 Department of Simulation Medicine, Faculty of Medicine, Masaryk University, Kamenice 5, 62500 Brno, Czech Republic; 6 Department of Pediatrics, University Hospital Brno, Černopolní 9, 61300 Brno, Czech Republic; 7 Department of Pediatric Neurology, University Hospital Brno, Černopolní 9, 61300 Brno, Czech Republic

**Keywords:** COVID-19, SARS-CoV-2, children, intensive care, ICU, MIS-C, isolation

## Abstract

Background: The proportion of intensive care unit (ICU) admissions in children that have and have not been directly caused by SARS-CoV-2 remains unclear. The aim of the study is to analyse a cohort of children admitted to the ICU with SARS-CoV-2 and determine whether the infection was the primary cause of their hospitalisation, a significant contributor, a suspected accomplice, or an incidental finding. Methods: This was a retrospective observational study of all the children admitted to the ICU with SARS-CoV-2 from March 2020 to February 2022 from the South Moravia region. The aim of the study was to assess whether the hospitalisation was likely to be directly caused by the virus (i.e., patients with acute COVID-19; the COVID group), whether the virus was a significant contributor to the hospitalisation (i.e., patients with multisystem inflammatory syndrome in children due to COVID-19; the MIS-C group), whether it may have contributed to the worsening of their underlying disease (the WORSENING group), or whether it was an incidental finding very likely unrelated to hospitalisation where SARS-CoV-2 positivity merely placed patients in the COVID-19 unit (the ISOLATION group). The groups were compared using a series of secondary outcomes. Results: The study population represented 150 paediatric ICU cases (age 8.6; IQR 3.5–13.3 years), with 66.7% being male. The COVID group represented 32.7% of cases (49/150); MIS-C, 30% (45/150); WORSENING, 14.7% (22/150); and ISOLATION, 22.7% (34/150). The median length of hospitalisation was found for the MIS-C group (11 days; 9 days in the ICU), the COVID group (6 days; five days in the ICU), WORSENING group (4.5 days; 4.5 days in the ICU) and the ISOLATION group (5.5 days; 3.5 days in the ICU), where the difference was significant (*p* < 0.001). Asymptomatic and mild cases were most common in the WORSENING (36.4% and 63.6%) and ISOLATION (52.9% and 44.1%) groups. Severe and critical cases were only present in the COVID (6.1% and 12.2%) and MIS-C (4.4% and 11.1%) groups; the severity difference was significant (*p* < 0.001). The groups did not differ significantly in the proportion of complete recovery and short- and long-term sequelae (*p* = 0.09). Conclusions: Patients with acute COVID-19 accounted for one-third of all ICU admissions, patients with MIS-C accounted for approximately another third, patients with worsening underlying disease accounted for 15%, and patients with incidental findings of SARS-CoV-2 positivity accounted for one-fifth of ICU admissions. A more significant disease was seen with acute COVID-19 and MIS-C.

## 1. Introduction

Although COVID-19 is predominantly a mild disease in children, during the pandemic, a proportion of children ended up in intensive care units (ICUs). One of the arguments for vaccinating children against COVID-19 is to protect them from severe courses that require intensive care [1].

In our country, there has been speculation about overestimated numbers of COVID-19 children in the ICU due to routine testing, which in turn has contributed to distrust of severe courses in children and, consequently, to the view that it is unnecessary to vaccinate children. 

The proportion of children in the ICU (from all admissions) ranged from 26.5–33.2% in the U.S. [2,3] and 18% in the U.K. [4]. However, some studies acknowledge the limitation that they can capture cases admitted to ICU where the virus was not the reason for hospitalisation [2,3,5]. An in-depth analysis is needed to evaluate the contribution of the virus to hospitalisation.

The aim of our study was to analyse a cohort of children admitted to the ICU with SARS-CoV-2 and determine whether the infection was the primary cause of their hospitalisation, a significant contributor, a suspected accomplice, or an incidental finding.

## 2. Materials and Methods

We analysed data from the central hospital, whose catchment area is the South Moravia region, which has 1.18 million inhabitants, including 247 thousand children (0–19 years). University Hospital Brno concentrated 95% of all hospitalised children with COVID-19 during the first two pandemics. All children from the region requiring intensive care were admitted to this hospital. 

The study population inclusion criteria included a diagnosis of SARS-CoV-2 infection, an age of 0–19 years, and hospitalisation in the intensive care unit at the University Hospital Brno from March 2020 to February 2022. 

Cases were retrospectively identified in the hospital database based on their final diagnosis at discharge (ICD-10 code U071). 

The database is not entirely reliable, as some uninfected patients in quarantine were given code U071 without any SARS-CoV-2 confirmation. Thus, we verified SARS-CoV-2 tests (PCR, antigen, and serology) by checking the medical record of each case to exclude any SARS-CoV-2-unrelated cases (the methods used to diagnose SARS-CoV-2 are described in detail in Supplementary File S1: Methods used in the study). We defined these cases as ICU hospitalisation without any SARS-CoV-2 direct or indirect confirmation in temporal relation to admission.

The database is also insufficient for identifying MIS-C cases (the ICD-10 classification of MIS-C was unclear initially). MIS-C cases were registered in our second data source, the database of MIS-C follow-ups. 

We also excluded rehospitalisations of the same case. Repeated admissions occurred in immunocompromised subjects with a prolonged positivity of SARS-CoV-2 who came for cancer treatment cycles. Technically, these were the same cases; therefore, we included only the patient’s first hospitalisation and discarded subsequent readmissions in the analysis. 

We had to omit all other uninfected patients in quarantine as we could not identify them. These cases were present only at the beginning of the pandemic and did not substantially contribute to the number of children in the ICU.

The primary outcome was the cause of hospitalisation in the ICU, which was evaluated and decided by the investigator based on the case history, course of the disease, and final diagnosis. 

Based on the cause, we divided patients into four groups: COVID, MIS-C, WORSENING, and ISOLATION.

COVID-19 patients were admitted due to acute SARS-CoV-2 infection. Acute infection was based on direct evidence of the virus and COVID-19 symptoms leading to admission. SARS-CoV-2 can be considered a primary cause of ICU hospitalisation in these cases. The criteria for inclusion in the COVID group were as follows:

- Newly developed symptoms: flu-like symptoms (fever, headache, sore throat, myalgia), clinical symptoms of respiratory (rhinorrhea, cough, dyspnea) or gastrointestinal infection ((nausea, vomiting, abdominal pain, diarrhoea), neurological symptoms (loss of smell or taste, febrile seizures), rash [6];
- Direct detection of SARS-CoV-2 by antigen test or RT-PCR from a nasopharyngeal swab during current illness [7];
- No other pathogen was identified that could explain the above symptoms.

MIS-C patients were admitted due to multisystem inflammatory syndrome in children with COVID-19 (MIS-C). In these cases, a SARS-CoV-2 infection can be considered an essential contributor alongside other not yet fully understood intrinsic host factors. The diagnosis of MIS-C was based on the CDC criteria:

- An individual aged <21 years presenting with fever, laboratory evidence of inflammation, and evidence of clinically severe illness requiring hospitalisation, with multisystem (>2) organ involvement (cardiac, renal, respiratory, haematologic, gastrointestinal, dermatologic, or neurological);
- No alternative plausible diagnoses;
- Positive for current or recent SARS-CoV-2 infection by RT-PCR, serology, or antigen test; or COVID-19 exposure within the 4 weeks prior to the onset of symptoms [8].

Patients in the WORSENING group were admitted because of the deterioration of their underlying disease. SARS-CoV-2 positivity placed them in a COVID-19 ward. SARS-CoV-2 may contribute to deterioration through various tissue involvement [9], but determining the extent of the impact of SARS-CoV-2 on exacerbation is difficult, especially when multiple pathogens have been confirmed. In these cases, the virus can be considered a suspected accomplice in worsening the underlying disease leading to ICU admission. The criteria for inclusion in the WORSENING group were as follows:

- A patient was admitted for an exacerbation of a chronic underlying condition (lasting at least one month)
  or
  admitted with another acute condition in which multiple causative agents have been identified and the problem, therefore, cannot be clearly attributed to a single one;
- Direct detection of SARS-CoV-2 by antigen test or RT-PCR from nasopharyngeal swab during a current illness.

ISOLATION patients were admitted for other reasons and were isolated because of SARS-CoV-2 positivity. The reason for admission was a condition unlikely to be related to the SARS-CoV-2 infection. Patients with an asymptomatic, nosocomial SARS-CoV-2 infection are also included in this group. In these cases, the contribution of SARS-CoV-2 to hospital admission is doubtful and can be considered an incidental finding. Criteria for inclusion in the ISOLATION group:

- A patient admitted for a condition likely unrelated to ongoing SARS-CoV-2 infection: elective procedures and treatments, surgery, trauma, intoxication, genetic diseases, clearly defined localised bacterial infections (e.g., urinary tract, eye, etc.) or patients hospitalised for other conditions in which SARS-CoV-2 infection has been evaluated as nosocomial;
- Direct detection of SARS-CoV-2 by antigen test or RT-PCR from nasopharyngeal swab during current illness.

The groups were further compared using secondary outcome measures. The null hypothesis was that all cases of SARS-CoV-2 infection in the ICU were similar. We hypothesised that the proposed groups had unique characteristics and that patients with acute COVID-19 or MIS-C or those in the WORSENING group might be more ill than those in the ISOLATION group.

The groups were compared based on sex, age, severity, length of hospital stay, ICU stay, recovery, laboratory signs of inflammation and cardiac damage, SARS-CoV-2 serology, cardiology, neurology, chest X-ray, brain MRI, and treatment (methods and instruments used are described in detail in Supplementary File S1: Methods used in the study.

Severity was assessed based on the previously published COVID-19 severity classification [10]. Asymptomatic disease is represented by SARS-CoV-2 positivity without symptoms. Mild disease is represented by a symptomatic course without dyspnoea, desaturation, and chest X-ray findings. Moderate disease is accompanied by dyspnoea, desaturation below 94%, and evidence of a lower respiratory tract infection. Severe disease is accompanied by desaturation below 94% and involvement of more than 50% of the lungs as identified through a chest X-ray. Critical illness is accompanied by organ failure or septic shock. However, the classification has its limits. There is no established severity classification for MIS-C disease. Similarly, it is difficult to classify the severity of heterogeneous conditions in the WORSENING and ISOLATION groups. In particular, the moderate and severe categories are inappropriate for diseases without a respiratory component. Knowing all these limitations and the lack of other tools, the COVID-19 classification was used for these other diseases to allow for a rough comparison between groups.

Length of hospitalisation: Patients were usually admitted to the ICU and continued their stay in a non-ICU ward. We evaluated the total hospital stay and how many days patients spent in the ICU.

Recovery: At discharge, we assessed healthy patients as fully recovered. In the case of patients with an underlying disease, complete recovery was a return to the original stable state before hospitalisation. Patients with an incomplete recovery were seen at follow-up visits. Follow-up included visits at 1–4 weeks, one, three and 12 months after discharge. Clinical examinations, laboratory tests, SARS-CoV-2 serology, cardiology, spirometry (children 6 years and older), and neurology (individualised by symptoms) were performed during visits. Patients with difficulties lasting less than one month were assessed as having short-term sequelae, and those with difficulties lasting more than one month as having long-term sequelae. No fatal cases were recorded; thus, we did not analyse any.

Laboratory signs of inflammation and cardiac damage: In most cases, C-reactive protein (CRP), fibrinogen, and D-dimers were available and selected as markers of inflammation for analysis. Troponin and N-terminal prohormone of brain natriuretic peptide (NTproBNP) were measured as signs of cardiac damage. The maximum values obtained were analysed.

Serology: We measured IgG against S and N antigens of SARS-CoV-2. The used quantitative method of antibody titre measurement varied during the analysed period; therefore, only the qualitative results were evaluated. Values above the cut-off were assessed as positive. Borderline and negative values were considered negative.

Cardiology: Cardiac examination included an electrocardiogram and cardiac ultrasound. The pathologies evaluated were arrhythmias, coronary artery enlargement, myocarditis, pericardial effusion, and their combination. We classified the presence of one or more pathological findings under the term cardiac complications.

Neurology: Neurological examination included a clinical evaluation and electroencephalogram. Pathological findings included impaired consciousness, neuropsychiatric symptoms, headache, meningeal syndrome, central paresis, flaccid paresis, haemorrhage, ischaemia, acute symptomatic seizures, vasculitis, brainstem involvement, and acute motor axonal neuropathy. All abnormal findings were included under the term neurological complications in this analysis.

Imaging methods: brain MRI and chest X-ray were evaluated. Both methods were used based on clinical indications. One radiologist on duty described the findings. Unclear findings were consulted and decided by the radiology clinic team.

Chest X-ray: We analysed the proportion of patients with pathological findings on chest X-ray images. The pathological findings were classified into a picture corresponding to bronchitis, pneumonia, or pneumonia with pleural effusion. The extent of lung involvement was assessed to determine the severity.

Magnetic resonance imaging (MRI) of the brain: We evaluated the proportion of patients with abnormal MRI findings. Abnormalities captured included brain plaque thickening, cytotoxic oedema, ventricular enlargement, postischaemic changes, cysts, brain atrophy, and extrapontine myelinolysis. All the above findings were included under the term brain MRI abnormality for analysis.

Treatment: We analysed the proportion of patients who needed treatments such as antibiotics, steroids, anticoagulation therapy (heparin or low-molecular-weight heparin), intravenous immunoglobulin therapy (IVIG), anti-SARS-CoV-2 monoclonal antibodies, vasopressors, oxygen administration, ventilation, and extracorporeal membrane oxygenation (ECMO).

Statistical analysis: Quantitative data in the set have an abnormal distribution and are described using the median and interquartile range (Q1–Q3). Absolute or relative frequencies represent categorical parameters. Pearson’s chi-squared test or Fisher’s exact test was used for testing the differences in categorical parameters, and the Kruskal–Wallis test was used for quantitative parameters. The tests are indicated in the text by a superscript (Pearson’s chi-squared test ^A^, Fisher’s exact test ^B^, and the Kruskal–Wallis test ^C^). We tested our hypotheses for a significance of *p* = 0.05 and used the SPSS Statistics 28 software for the analysis.

## 3. Results

### 3.1. Population

The national database registered 88.558 SARS-CoV-2-positive children in the area during the first two years of the pandemic (two-year cumulative incidence, 35.790/100 k). Of them, 422 were hospitalised (170/100 k), and 95% (400/422) were identified by ICD-10 code U071 in the University Hospital Brno database. Additionally, 34 MIS-C subjects were not recognised by the code but were found in the MIS-C follow-up database. We excluded eight cases unrelated to SARS-CoV-2 and eight readmissions of the same case. This selection constitutes 419 unique hospitalisations of children with SARS-CoV-2. Of them, 269 patients were treated in non-ICU wards, and 150 were in the ICU. These cases comprise the study population.

**Figure 1:**
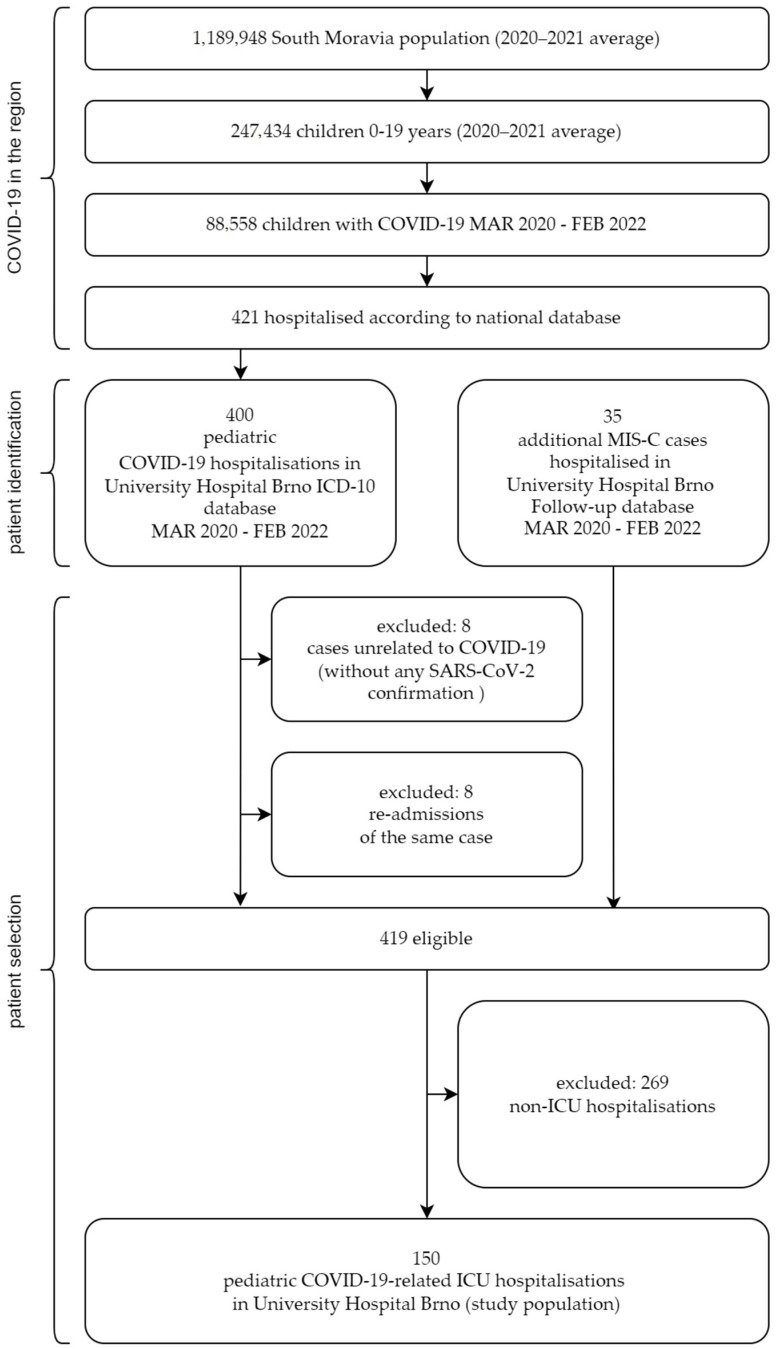
Patient identification and selection.

The identification and selection process of this study population is shown in Figure 1.

### 3.2. Demographics and Groups

In the study population, 66.7% were male, and the median age was 8.6 (IQR 3.5–13.3) years. After evaluating the cause of hospitalisation, the study population was divided into four groups: COVID, MIS-C, WORSENING, and ISOLATION. The COVID and MIS-C groups each represent almost one-third of the patients, The ISOLATION group accounts for one-fifth, and the WORSENING group accounts for less than 15%. A detailed list of diagnoses in the groups can be found in Supplementary Table S1: Groups and basic characteristics. A. The groups did not differ significantly by gender (*p* = 0.374) ^A^ or age (*p* = 0.594) ^C^; males dominated all groups. Demographic data of the study population and each group are shown in Table 1.

### 3.3. Comparison of Groups

The basic parameters for comparing the groups were severity, length of hospital and ICU stay, and recovery, which are shown in Table 2.

#### 3.3.1. Severity

Asymptomatic cases were present only in the WORSENING (36.4%) and ISOLATION (52.9%) groups. Mild cases were the most common in all groups (MIS-C, 82.2%; WORSENING, 63.6%; COVID, 59.2%; and ISOLATION, 52.9%). Moderate cases were significantly represented only in the COVID group (22.4%). Severe and critical cases were present only in the COVID and MIS-C groups (12.2% and 11.1%, respectively). The difference in severity between groups was significant (*p* < 0.001).

#### 3.3.2. Length of Hospitalisation

Patients in the MIS-C group spent the longest median time in the hospital and ICU (11 and 8 days, respectively). Patients in the COVID and ISOLATION groups spent a similar amount of time in the hospital (6 and 5 days, respectively). Patients in the COVID group stayed in the ICU longer (5 days). Patients in the WORSENING group were hospitalised for the shortest time (4.5 days). The time spent in the hospital and the ICU differed significantly between the groups (*p* < 0.001).

#### 3.3.3. Recovery

In all groups, complete recovery was predominant (WORSENING, 95.5%; ISOLATION, 85.3%; MIS-C, 80%; and COVID, 69.4%). Short-term sequelae were similarly represented in the COVID, MIS-C, and ISOLATION groups (16.3%, 15.6%, and 14.7%, respectively). Long-term sequelae were represented more in the COVID group (14.3%). The differences in recovery between the groups were not significant (*p* = 0.09).

Laboratory tests, imaging, and complementary examinations were used to compare the groups in more detail. The results are shown in Table 3.

#### 3.3.4. Laboratory Tests

The CRP was available in almost all patients (144/150). It was significantly highest in the MIS-C group (180.2 mg/L) and low in the other groups (*p* < 0.001). Fibrinogen was examined in 104/150 patients. It was also significantly highest in MIS-C patients (5.9 g/L).

Troponin and NTproBPN results were available mainly from the MIS-C group (n = 43/45), where they were the highest. They were rarely examined in the COVID and WORSENING groups (8/49 and 3/22, respectively). They were not examined at all in the ISOLATION group. Differences between groups were significant only for NTproBPN (*p* = 0.025).

D-dimers were tested more frequently (104/150) and were again highest in the MIS-C group (3.8 ng/mL FEU). The difference in D-dimers between groups was significant (*p* < 0.001).

Antibodies to S and N antigens were predominantly present in the MIS-C group (86.7% and 77.3%, respectively), which was a significant difference from the other groups where positivity was low (*p* < 0.001).

#### 3.3.5. Brain MRI

MRI of the brain was rarely performed (8/150 patients), and abnormal findings were present only in 3 cases. Patients with abnormal findings were in the COVID and MIS-C groups, and the differences were insignificant.

#### 3.3.6. Chest X-ray

The number of patients with chest X-ray findings consistent with bronchitis was highest in the MIS-C group (44.5%). In contrast, pneumonia was most present in the COVID group (24.5%). Pleural effusion was represented in the COVID and MIS-C groups (10.2% and 8.9%, respectively). In the WORSENING and ISOLATION groups, abnormalities were rarely demonstrated on X-ray images. The groups differed significantly in radiographic findings (*p* < 0.001).

#### 3.3.7. Cardiac Complications

A total of 30 patients in the study population had cardiac complications. The most common types of cardiac involvement were myocarditis (12/30), arrhythmia (6/30), pericardial effusion (5/30), or a combination of these (9/30). Coronary involvement was noted in only one patient. Cardiac complications occurred mainly in the MIS-C group (55.6%) and partially in the COVID (10.1%) and WORSENING (13.6%) groups. No cardiac complications occurred in the ISOLATION group. Differences between groups in cardiac complications were significant (*p* < 0.001).

#### 3.3.8. Neurological Complications

Neurological complications were reported in less than one-tenth of all patients (12/150). The most frequently reported pathology was seizures (8/12). Neurological complications occurred in all groups, with the highest incidence in the COVID group (10.2%), but the differences were insignificant (*p* = 0.885).

Finally, the groups were compared according to the treatment used. The comparison is shown in Table 4.

#### 3.3.9. Treatment

Significant differences between groups were noted in regard to antibiotics, corticosteroids, anticoagulation, IVIG, and oxygen administration.

Antibiotics were used in all MIS-C cases (100%) and half of the other groups’ cases. Steroids were administered to almost all MIS-C patients (93.3%) and half of the COVID-19 patients (49%). Anticoagulation therapy was needed in MIS-C patients (93.3%) and a quarter of COVID-19 patients (24.5%). IVIG was almost exclusively used in the MIS-C group (86.7%). Oxygen therapy was needed most by patients in the COVID group (36.7%).

Differences between groups in regard to vasopressors, monoclonal antibodies, and ventilatory support were insignificant. These therapies were used in fewer patients (vasopressors, 7/150; monoclonal antibodies, 9/150; and ventilatory support, 6/150).

Ventilation was required in five patients in the COVID group, and the median duration was 14 days. Only one patient with MIS-C was on ventilation for six days. No patient required ECMO.

## 4. Discussion

The demographics of our study population, with a median age of 8.6 years, are very similar to previously published data (8.9 years [4] to 9.7 years [7]). Still, our study population’s median age was younger than that of the pandemic’s early phase (13 years) [11].

As in our cohort (66.7%), a male preponderance is published throughout other studies but is usually less pronounced [2,3,11,12,13,14,15,16]. The prevalence of boys is more pronounced in MIS-C (59–65%) [17].

Depending on the period, the proportion of children admitted to the ICU ranged from 26.5–33.2% [2,5]. In a prospective U.K. study, it was less (18%) [4]. In our study cohort, it was 35% (150/419).

Regarding the assessment of the cause of admission, most studies do not usually differentiate between them and describe the entire cohort of patients admitted with COVID-19 [2,18]. Some studies distinguish between the acute COVID-19 and MIS-C groups, which they then compare [4,11]. A few studies single out a group of hospitalised children whose admissions are unlikely to be related to SARS-CoV-2. Two studies, one from Norway and the other from South Africa, adopted a similar methodology to our study to assess the causes of hospital admissions in children with COVID-19 [19,20].

The South African study analysed all children hospitalised from one region (Tswane) during the Omicron wave [19]. Available data allowed for an in-depth analysis. Based on the assessment of diagnosis at discharge, the study concluded that 44% (61/138) had COVID-19 as the primary diagnosis, 20% (27/138) had it as the contributory diagnosis, and 36% (27/138) had it as the incidental diagnosis. No patient had a diagnosis of MIS-C. However, this analysis includes both ICU and non-ICU children. Unfortunately, detailed data were only available for a proportion of all children admitted (138/183). The study focused on a short period of 5 weeks around November 2021; therefore, a comparison with our results is limited.

The Norwegian study compared the risks of hospitalisation depending on the virus variant [20]. Norway adopted a similar testing strategy as our country (children were regularly tested twice a week in schools, etc.). The study covered a similar period as our analysis (March 2020–January 2022). During the mentioned period, 174 children were hospitalised with positive SARS-CoV-2. The primary cause of hospitalisation was COVID-19 in 55% (97/174) and MIS-C in 16% of patients (28/174). The rest, 28% (49/174), were not identified. The median length of hospitalisation for acute COVID-19 and MIS-C was 0.8–1.4 days and 4 days, respectively, which is significantly less than in our study. Over the entire period of the Norwegian study, only 6% of acute COVID-19 and 14% of MIS-C cases required ICU hospitalisation. Given the similar testing system, comparing our results with those of this study would be beneficial. However, the authors did not comment on the reasons for hospitalisations in nearly one-third of the patients. In addition, they acknowledge the unusually low numbers of MIS-C and ICU cases compared with other studies.

In the “Risk Factors for Severe COVID-19 in Children” study, the authors evaluated 3106 paediatric hospitalisations with positive SARS-CoV-2 [12]. Of these, 73% (2293/3106) were primary admissions for COVID-19. In the authors’ assessment, 23% (718/3016) of patients who had SARS-CoV-2 but were not admitted for COVID-19 were excluded from the evaluation (170 were admitted to the ICU). Their characteristics can be found in the Supplementary Table S1. Reasons for hospitalisation were delivery, 19.9%; surgery or procedure, 14.5%; psychiatric admissions, 33.2%; trauma, 16.7%; and other, 11.3%, which are similar cases that would fall into the ISOLATION group in our analysis.

In assessing the causes of hospitalisation, we identified four distinct groups (COVID, MIS-C, WORSENING and ISOLATION). In each of them, SARS-CoV-2 contributed differently to the hospitalisation.

Patients with acute COVID-19 (the COVID group) accounted for 32.7% of cases. In other studies, the proportion of acute COVID-19 varies significantly by design, from 51.7% to 88.6% [4,7].

Disease severity in the COVID group included all categories from mild to critical. Most studies assessed severity according to the same or slightly modified classification [10,11,18,21]. The proportion of severities varied considerably. In a study of ICU admissions from the beginning of the pandemic, the proportion of asymptomatic and critical cases was 29% and 35%, respectively [11]. The proportion was 0% asymptomatic and 12.2% critical cases in our COVID group.

ICU stay in the COVID group was 5 days, similar to the overall ICU cohort (5 days) [11] and severe COVID-19 cohort (4 days) [7]. In studies that include ICU and non-ICU, hospitalisation tends to be shorter, e.g., 2 days in South Africa [19], 2–3 days in a U.S. cohort [2], and 5 days in a Polish study [22].

Complete recovery at the time of discharge occurred in 69% of patients in the COVID group. Long-term sequelae were present in 14%. Sequelae present after the disease were addressed in the long COVID studies [23]. The definition of long COVID is not settled. Still, it is usually assessed as the persistence of symptoms for more than 4 weeks corresponding to the long sequelae identified in our study. The proportion of persisting sequelae was usually around 4–9% in studies with clinical follow-up [24,25], slightly less than our COVID group (14.3%).

Patients in the COVID group generally had lower signs of inflammation (CRP 12.2 mg/L), which is consistent with the majority of published data [7,19,22] which also applies to the incidence of pneumonia and pleural effusions in the COVID group [4,15,18,22].

Cardiac and neurological complications occurred in 10.2% of the COVID group. Similar to our findings, cardiac complications were observed in 11.8% of severe acute COVID-19 cases [7]. On the other hand, neurological complications were more frequently observed in acute severe COVID-19 (19.9%) [7], which is different from our study.

Patients in the COVID group had the highest use of oxygen therapy (36.7%). Antibiotics (53.1%), steroids (49%), and anticoagulation therapy (24.5%) were frequently used. In a U.K. prospective study on COVID-19, antibiotics were used in 63.7% of cases [4] and oxygen was administered to 26.4% of patients and high-flow nasal cannula oxygen was administered to 11.7% (38.1% in total), which is similar to our COVID group (36%). Steroids were in acute COVID-19 (28.1%) patients. IVIG was administered to MIS-C patients in 77% of cases and acute COVID-19 patients in 4.2% of cases [4,7].

Patients in the MIS-C group accounted for 30% of all cases. The proportion of MIS-C varies significantly by study design, from 11.2–49.5% [4,7]. Disease severity in the MIS-C group also included all categories except asymptomatic, but more mild cases were recorded compared to other groups. The high proportion of mild cases is probably related to the low proportion of patients with desaturation and the need for oxygen therapy, which would classify the cases into higher severities. The proportion of critical cases (11.1%) was similar to that of the COVID group. Other studies usually did not measure the severity of MIS-C.

The median length of hospital stay was 11 days, including nine days in the ICU, the longest out of all groups. According to published data, the median length of MIS-C hospitalisation was 5–7 days (4 days in the ICU) [7,26]), and according to a review of different case series, the mean hospital stay was 7 days (standard deviation 11 days) [27].

Complete recovery occurred in 80% of patients at discharge, with long-term sequelae present in 4.4%. This is less than in the MIS-C follow-up study, where 47% of patients reported difficulties 2 weeks after discharge [26]. Within 8 weeks, the number of patients with complaints dropped to 12%. Coronary involvement was evident in 12%. By 6 months, the discomfort had resolved in all patients.

Patients in the MIS-C group had the highest signs of inflammation (CRP 180.2 mg/L) and cardiac damage (troponin 23.5 ng/L and NTproBNP 2646.0 ng/L), which is consistent with published data [7,19,22,26,27,28]. Patients with MIS-C had the most frequent positive serologies against S and N antigens out of all the groups (86.7% and 77.3%, respectively). Bronchitis was the predominant picture on chest X-ray images (44.4%).

Cardiac complications were the most frequent in this group (55.6%). Cardiac involvement is reported in about 66.7% to 80% of MIS-C cases [7,17].

Neurological complications were observed in 6.7% of cases in the MIS-C group, which is less than published (12.2%) [7]. In a review study of patients with severe neurological complications, 40% (65/159) had MIS-C [29].

Patients with MIS-C required the most intensive treatment. Antibiotics were administered in 100% of cases, steroids in 93.3%, anticoagulation in 93.3%, and IVIG in 86.7%. Oxygen therapy was needed in 11.1% of cases. The frequent use of antibiotics in our MIS-C group (100%) is based on internal hospital guidelines, which recommend that patients with sepsis be provided with antibiotics until stabilisation and refinement of the diagnosis. According to published data, steroids were administered to MIS-C in 46.2–66.9% and IVIG in 77% of cases [4,7].

Data from the WORSENING and ISOLATION groups are difficult to compare with published data because, to our knowledge, other studies do not evaluate these patients separately. They can be compared with our COVID and MIS-C groups

Patients with a worsening underlying disease in the WORSENING group accounted for 14.7% of all cases. Patients had only asymptomatic or mild courses of infection. The median length of hospital and ICU stay was 4.5 days. In this group, most subjects had a complete recovery (95.5%), and no long-term sequelae were present. Signs of cardiac damage and inflammation were low (CRP 8.1 mg/L). Patients in the WORSENING group were almost free of abnormal findings on chest X-ray images. Cardiac and neurological complications were similar to those in the COVID group (13.6% and 9.1%, respectively). Antibiotics (50%) and steroids (18.1%) were the most frequently administered treatment. Patients in this group did not require oxygen therapy or ventilatory support.

Patients in the ISOLATION group accounted for 22.7% of cases. Patients had only asymptomatic or mild courses of infection. The median length of hospital stay was 5.5 days. These patients spent the shortest time in the ICU (3.5 days). Complete recovery occurred in 85.3% of patients. Signs of inflammation markers were the lowest in this group (CRP 3.8 mg/L). Abnormal findings on chest X-ray images were rare. Patients in the ISOLATION group had no cardiac complications. Treatment in the ISOLATION group was similar to that in the WORSENING group, i.e., antibiotics were used in 52.9% of cases and steroids in 15.2%. Only one patient needed oxygen therapy.

This study was possible due to the strength of the study population. The University Hospital Brno is in a similar situation as other central hospitals in that all hospitalised children with the infection from the region are concentrated here. Tertiary hospitals in our region hospitalise children with COVID-19 at a minimal rate (5%); thus, we had data on virtually all hospitalised children in the region over two years. We seized the opportunity to analyse the population of children in the ICU without much concern about selection bias.

During this period, there was a regulation for the universal testing of children twice a week in schools and mandatory testing before hospital admission for COVID-19. Thus, the detection of SARS-CoV-2 was independent of a clinical indication. The above significantly reduced the risk of selection bias as we captured almost all inpatients and ICU cases from the region’s population.

Another strength was the one-year follow-up system for patients with problems after COVID-19, which allowed for the assessment of sequelae.

The study had several limitations. First, because this is a single-centre study, our results may not apply to the entire population.

Second, the study was narrowly focused on ICU admissions only. Because many of the SARS-CoV-2 infections in children are mild and these children did not require hospital care, our results cannot be applied to the entire population. However, the results may apply well to children with the infection who need intensive care.

Third, some groups include a small number of cases (especially the WORSENING group), which may limit the assessment of the impact of SARS-CoV-2 on the cause of hospitalisation. However, this is offset by the high probability that we captured all cases from the catchment area in a given group.

Fourth, the assessment of disease severity was based on the classification for COVID-19. The classification focuses on the respiratory component of the disease and does not ideally capture the broader range of symptoms (e.g., dehydration from diarrhoea) that can occur in children. MIS-C usually presents a severe picture, but most cases fall into the mild category due to the absence of desaturations. Similarly, severity ratings were limited in the WORSENING and ISOLATION groups. However, a rough picture of the prevalence of asymptomatic cases in both groups can be drawn from the comparison.

Next, the ICD-10 database that includes discharge diagnoses is not entirely reliable due to the unclear classification at the beginning of the pandemic, when codes for COVID-19 were assigned to uninfected patients in quarantine. MIS-C coding was similarly ambiguous. All classified cases were manually checked so that redundant cases were discarded. The unreliability of the database may cause some hospitalised patients not to be identified. However, given the similarity of national and hospital data, it is likely that only a few individuals may have been involved.

Finally, we could not assess the impact of vaccination available to children in the second half of the evaluation period. However, childhood vaccination rates against COVID-19 are persistently low in our country (0–5 years 0%, 5–12 years 6.4%, 12–19 years 38.8%).

## 5. Conclusions

To our knowledge, this is the first study to evaluate the causes of ICU admission in children with SARS-CoV-2 infection, including an analysis of cases where the virus contributed little or no evidence at all of the disease.

SARS-CoV-2 was the primary cause of admission in one-third of cases with acute COVID-19. In another third of cases, the virus was a significant contributor to MIS-C. In 15% of admissions, the virus may have aggravated the underlying disease. In one-fifth of the cases, viral infection could be considered an incidental finding that led only to patient isolation. This fact should be kept in mind when returning to routine testing before hospital admission.

## Figures and Tables

**Table 1:** Demographics.

	Study Population	COVID	MIS-C	WORSENING	ISOLATION
Cases	150	32.70% (49/150)	30.0% (45/150)	14.7% (22/150)	22.7% (34/150)
Sex, male	66.7% (100/150)	66.3% (33/49)	75.6% (34/45)	59.1% (13/22)	58.8% (20/34)
Age, years (IQR)	8.6 (3.5–13.3)	6.1 (1.3–14.5)	10.1 (7.4–11.7)	7.0 (3.0–14.5)	9.8 (3.5–14.3)

**Table 2:** Severity, length of hospitalisation, and recovery.

	COVID	MIS-C	WORSENING	ISOLATION	*p*-Value
Severity					*p* < 0.001 ^B^
Asymptomatic	0%	0%	36.4% (8/22)	52.9% (18/34)	
Mild	59.2% (29/49)	82.2% (37/45)	63.6% (14/22)	44.1% (15/34)	
Medium	22.4% (11/49)	2.2% (1/45)	0%	2.9% (1/34)	
Severe	6.1% (3/49)	4.4% (2/45)	0%	0%	
Critical	12.2% (6/49)	11.1% (5/45)	0%	0%	
Hospital stay, days (IQR) ICU stay, days (IQR)	6.0 (4.0–11.0) 5.0 (3.0–10.0)	11.0 (9.0–13.0) 9.0 (7.0–11.0)	4.5 (3.0–10.0) 4.5 (3.0–10.0)	5.5 (2.0–10.0) 3.5 (2.0–7.0)	*p* < 0.001 ^C^ *p* < 0.001 ^C^
Recovery					*p* = 0.09 ^B^
Complete Short sequelae Long sequelae	69.4% (34/49) 16.3% (8/49) 14.3% (7/49)	80.0% (36/45) 15.6% (7/45) 4.4% (2/45)	95.5% (21/22) 4.5% (1/22) 0%	85.3% (29/34) 14.7% (5/34) 0%	

^B^ Fisher’s exact test; ^C^ Kruskal–Wallis test.

**Table 3:** Laboratory tests, imaging, and complementary examinations.

	COVID	MIS-C	WORSENING	ISOLATION	*p*-Value
Laboratory tests					
CRP, mg/L (IQR)	12.2 (5.4–73.6)	180.2 (106.5–230.6)	8.1 (1.0–53.5)	3.8 (1.3–76.3)	*p* < 0.001 ^C^
Fibrinogen, g/L (IQR)	4.0 (2.7–5.7)	5.9 (5.0–7.3)	2.7 (2.2–3.7)	3.4 (2.5–4.1)	*p* < 0.001 ^C^
Troponin, ng/L (IQR)	19.0 (3.0–43.0)	23.5 (6.0–47.0)	4.0 (0.0–17.0)	-	*p* = 0.210 ^C^
NTproBNP, ng/L (IQR)	365.5 (18.5–11,099.0)	2646.0 (888.0–8843.0)	303.5 (10.7–865.5)	-	*p* = 0.025 ^C^
D-dimer, ng/mL FEU (IQR)	1.6 (0.7–3.5)	3.8 (2.2–5.6)	0.7 (0.3–2.3)	0.8 (0.3–2.3)	*p* < 0.001 ^C^
COVID IgG anti-S positive	12.8% (6/49)	86.7% (39/45)	13.6% (3/22)	17.6% (6/34)	*p* < 0.001 ^B^
COVID IgG anti-N positive	4.1% (2/49)	77.3% (34/45)	9.1% (2/22)	14.7% (5/34)	*p* < 0.001 ^B^
Imaging					
Brain MRI pathology	6.3% (3/49)	2.3% (1/49)	0%	0%	*p* = 0.207 ^B^
X-ray bronchitis Pneumonia Pleural effusion	14.3% (7/49) 24.5% (12/49) 10.2% (5/49)	44.4% (20/45) 4.4% (2/45) 8.9% (4/45)	9.1% (2/22) 4.5% (1/22) 0%	9.1% (3/34) 0% 3% (1/34)	*p* < 0.001 ^B^
Complementary examinations					
Cardiac complications	10.2% (5/49)	55.6% (25/45)	13.6% (3/22)	0%	*p* < 0.001 ^B^
Neurological complications	10.2% (5/49)	6.7% (3/45)	9.1% (2/22)	5.9% (2/34)	*p* = 0.885 ^B^

^B^ Fisher’s exact test; ^C^ Kruskal–Wallis test.

**Table 4:** Treatment.

	COVID	MIS-C	WORSENING	ISOLATION	*p*-Value
Antibiotics	53.1 % (26/49)	100.0 % (45/45)	50% (11/22)	52.9% (18/34)	*p* < 0.001 ^A^
Steroids	49.0% (24/49)	93.3% (42/45)	18.1% (4/22)	15.2% (5/34)	*p* < 0.001 ^B^
Anticoagulation therapy	24.5% (12/49)	93.3% (42/45)	9.1% (2/22)	8.8% (3/34)	*p* < 0.001 ^A^
IVIG	6.1% (3/49)	86.7% (39/45)	4.5% (1/22)	5.9% (2/34)	*p* < 0.001 ^A^
Monoclonal antibodies	6.1% (3/49)	6.7% (3/45)	9.1% (2/22)	2.9% (1/34)	*p* = 0.767 ^B^
Vasopressors	8.2% (4/49)	15.6% (7/45)	0%	2.9% (1/34)	*p* = 0.116 ^B^
Oxygen therapy	36.7% (18/49)	11.1% (5/45)	0%	2.9% (1/34)	*p* < 0.001 ^B^
Ventilation	10.2% (5/49)	2.2% (1/45)	0%	0%	*p* = 0.101 ^B^
Ventilation length, days (IQR) ECMO	14.0 (7.0–18.0) 0%	6.0 (6.0–6.0) 0%	0.0 0%	0.0 0%	*p* = 0.380 ^C^ ^-^

^A^ Pearson’s chi-squared test; ^B^ Fisher’s exact test; ^C^ Kruskal–Wallis test.

## Data Availability

The data presented in this study are available on request from the corresponding author. The data are not publicly available due to ethical restrictions.

## Supplementary Materials

The following supporting information can be downloaded at: https://www.mdpi.com/article/10.3390/children10010075/s1. Table S1: Groups and basic characteristics; File S1: Methods used in the study.
