# Supplementary material for: Causes of Intensive Care Unit Admissions in Children with SARS-CoV-2: A Single-Centre Observational Study
Source: Children (Basel). 2022 Dec 30;10(1):75. doi: 10.3390/children10010075 (PMC9856882; doi:10.3390/children10010075)
Supplement: Supplementary file 1 [file children-10-00075-s001.zip › File S1 Methods used in the study.pdf]

children

Supplementary Materials

Methods used in the study

The following are the methods used to diagnose and evaluate the hospitalized patients in the study. All methods were used during routine clinical practice.

1. SARS-CoV-2 identification

1.1. Real time polymerase chain reaction

Method A

Predominated before 2022

Isolation:

Kit: DB-1206 Automated RNA isolation kit for Agilent Bravo (DIANA Biotechnologies s.r.o., Czech Republic)

Robot: Agilent Bravo

Detection:

Kit: DB-1211 COVID-19 Multiplex RT-PCR Kit (DIANA Biotechnologies s.r.o., Czech Republic);(multiplex format detection of Spike and EndoRNAse)

Cycler: BIO-RAD CFX96

Software: CFX Maestro Software Version 2.0.

Method B

Predominated from 2022

Kit: Alinity m SARS-CoV-2 assay (dual target assay RdRp aand N)

Analysator: Alinity m (Abbott Molecular Inc., Illinois, IL, USA)

Method C

Used for rapid diagnostics

Kit: Cobas® SARS-CoV-2 & Influenza A/B test

Analysator: Cobas® Liat® System

1.2. Rapid antigen test

Abbott PANBIO™ COVID-19 Ag RAPID TEST DEVICE

1.3. Serology SARS-CoV-2

1.4. Analysator: Cobas Roche®

1.4.1. IgG anti S: U/mL, positive above 0.8

1.4.2. IgG anti N: COI, positive above 1.0

2. Imaging methods **- KDN**

2.1. Brain magnetic resonance

MR je Siemens Aera 1.5T

standard protocol: T2 axial, T1 sagittal, axial, coronary, FLAIR axial, DWI/ADC, SWI, T1 C+ axial, coronary

2.2. Chest X-Ray

GE Definium 8000 or Siemens Ysio

standard protocol: front to back image

3. Cardiology
3.1. Electrocardiogram: BTL–08 LC
3.2. Heart ultrasound: VIVID E95 (GE)

4. Neurology
4.1. Electroecephalogram

Model: EEG TruScan CL 32

Routine EEG protocol: 20minute-rest state with closed eyes, reaction to eyes opening, photostimulation, mouth and nose hypervetilation

5. Laboratory tests
5.1. C-reactive protein

Immunoturbidimetry
Ref. range: 0,00 – 5.00 mg/L

5.2. Fibrinogen

Clauss clotting method
Ref. range :
0 – 1 year 1,5 - 3,4 g/L
1 - 6 years 1,7 - 4,0 g/L
6 – 11 years 1,55- 4,0 g/L
11 - 16 years 1,55 - 4,5 g/L
16 - 18 years 1,6 - 4,2 g/L
above 18 1,8 - 4,2 g/L

5.3. D-dimer FEU

Immunoturbidimetry
Ref. range: 0,00 – 0,50 mg/L

5.4. Troponin

Electrochemiluminescence immunoassay (ECLIA)
Cutt off: 14 ng/L

5.5. N-terminal prohormone of brain natriuretic peptide

Electrochemiluminescence immunoassay (ECLIA)
Cutt off acute heart failure: 300 ng/L
Cutt off chronic heart failure: 125 ng/L
